# Extracoporeal photopheresis treatment of acute graft-versus-host disease following allogeneic haematopoietic stem cell transplantation

**DOI:** 10.12688/f1000research.8118.1

**Published:** 2016-06-27

**Authors:** Aisling M. Flinn, Andrew R. Gennery

**Affiliations:** 1 Primary Immunodeficiency Group, Institute of Cellular Medicine, Newcastle University, Newcastle upon Tyne, UK; 2 Paediatric Haematopoietic Stem Cell Unit, Great North Children’s Hospital, Newcastle upon Tyne, UK

**Keywords:** Acute graft-versus-host disease, Acute graft-versus-host disease, stem cell transplantation, aGvHD, Extracorporeal photopheresis

## Abstract

Acute graft-versus-host disease (aGvHD) continues to be a major obstacle to allogeneic haematopoietic stem cell transplantation. Thymic damage secondary to aGvHD along with corticosteroids and other non-selective T lymphocyte-suppressive agents used in the treatment of aGvHD concurrently impair thymopoiesis and negatively impact on immunoreconstitution of the adaptive immune compartment and ultimately adversely affect clinical outcome. Extracorporeal photopheresis (ECP) is an alternative therapeutic strategy that appears to act in an immunomodulatory fashion, potentially involving regulatory T lymphocytes and dendritic cells. By promoting immune tolerance and simultaneously avoiding systemic immunosuppression, ECP could reduce aGvHD and enable a reduction in other immunosuppression, allowing thymic recovery, restoration of normal T lymphopoiesis, and complete immunoreconstitution with improved clinical outcome. Although the safety and efficacy of ECP has been demonstrated, further randomised controlled studies are needed as well as elucidation of the underlying mechanisms responsible and the effect of ECP on thymic recovery.

## Introduction

Allogeneic haematopoietic stem cell transplantation (HSCT) is used to treat malignant and non-malignant haematological conditions ^1^. In primary immunodeficiency, the aim following HSCT is to achieve complete and long-lasting immunoreconstitution (IR) with a diverse T cell receptor (TCR) repertoire, providing adequate adaptive T lymphocyte immunity ^2^. Delayed or persisting immunodeficiency is associated with significant morbidity and mortality with increased risk of infection, relapse, and development of secondary malignancies ^3, 4^. Potential strategies to boost thymic function and promote faster and complete IR, particularly in older patients who exhibit reduced thymic function inherently due to aging, have garnered much interest to improve patient outcome. Such approaches include the use of Fgf7 or sex steroid hormone inhibition, which have been shown to protect thymic epithelial cells (TECs) and improve thymopoiesis in experimental models ^5^.

## Effect of graft-versus-host disease on T lymphocyte immunoreconstitution

Conditioning given prior to HSCT results in an inevitable period of aplasia with obliteration of innate and adaptive immune responses, subjecting the patient to a period of increased risk of infection and other complications until the stem cells engraft and reconstitution of the immune system compartments ensues. Rebuilding of innate immunity, including monocytes, granulocytes, and epithelial barriers, occurs relatively quickly following HSCT, providing protection against bacterial and fungal infections ^6^. In contrast, T lymphocyte reconstitution is lengthier and more complex, involving two pathways ^6– 8^. Peripheral thymic-independent expansion of surviving host T lymphocytes and/or transferred donor T lymphocytes provides a degree of immediate T lymphocyte immunity but of limited diversity and permanency ^5^. Complete IR following lymphodepletion requires durable *de novo* thymic regeneration of naïve T lymphocytes from donor progenitor cells with a broad TCR repertoire, which requires a functioning and structurally intact thymus ^8, 9^. These naïve T lymphocytes (termed recent thymic emigrants [RTEs]) can be measured quantitatively by identification of surface markers such as CD45RA and CD31 using flow cytometry and by determination of TCR excision circle (TREC) levels. TRECs are circular pieces of DNA produced as a consequence of TCR α and β chain formation, and quantification of TREC content in T lymphocytes provides a practical and accepted measurement of thymic output ^10^. The quality of the T lymphocyte compartment can be assessed by measuring TCR diversity, as this is almost completely reflective of the naïve T lymphocyte population ^11^. This can be done using flow cytometry, spectratyping of the complementarity determining region 3 (CDR3), and nucleotide sequencing. Flow cytometry is widely available and cheaper and results can be obtained quickly ^12^. Spectratyping analyses the lengths of the hypervariable region CDR3 in each Vβ family using real-time polymerase chain reaction ^13, 14^. Compared to flow cytometry, spectratyping provides more detailed resolution of TCR diversity; however, there is no accepted single standardised method of analysing data at present, and this technique gives equal weighting to all Vβ families measured, independent of how many genes they contain ^13^. Nucleotide sequencing of DNA CDR3 regions provides even more in-depth analysis but is expensive and, although evolving, is not widely available at present ^15^. Thymic damage disrupts normal T lymphocyte ontogeny, resulting in reduced export of RTEs and a distorted TCR repertoire, negatively impacting on IR and clinical outcome ^5, 16– 18^.

Graft-versus-host disease (GvHD) is a leading cause of post-HSCT mortality ^19, 20^. Acute (a)GvHD is mediated by alloreactive mature donor T lymphocytes, which attack disparate recipient antigens, resulting in a harmful inflammatory response and tissue injury ^21^. Elucidation of aGvHD pathophysiology is based on experimental models ^20^: (1) damage to host tissue by conditioning regimens, underlying disease, and/or infections increases pro-inflammatory cytokines activating host antigen-presenting cells (APCs); (2) donor T lymphocytes recognise the disparate alloantigens on activated host APCs and become activated, proliferate, differentiate, produce further inflammatory cytokines, and migrate to target organs; (3) effector cells, primarily cytotoxic T lymphocytes and natural killer (NK) cells, and soluble effectors cause apoptosis of target cells.

**Figure 1.**
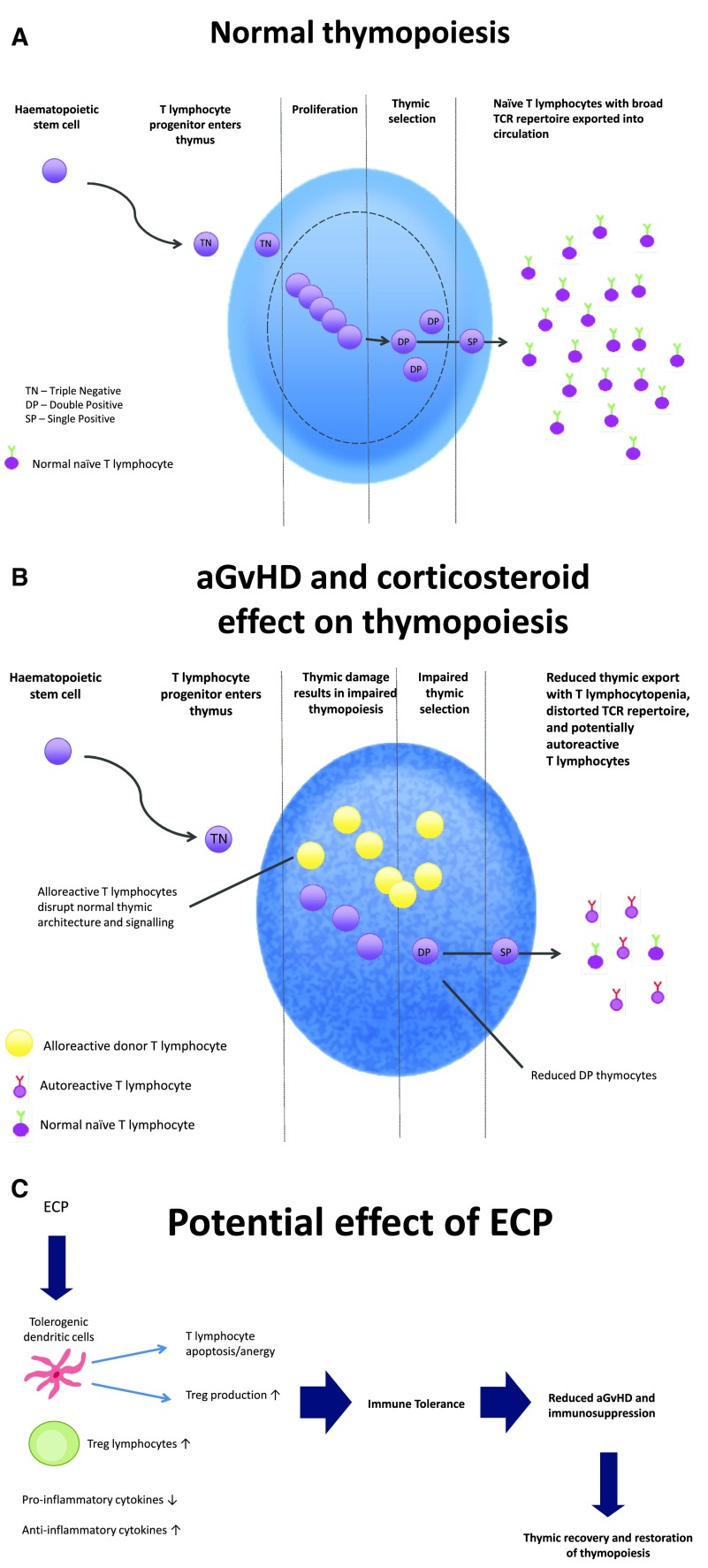
Normal thymopoiesis, effect of acute graft-versus-host disease (aGvHD) and corticosteroids on thymic function, and the potential effect of extracorporeal photopheresis (ECP) allowing thymic recovery. Thymic damage occurs secondary to allogeneic T lymphocytotoxicity during aGvHD, corticosteroid-mediated damage, and other non-selective T lymphocyte-suppressive agents used in the treatment of aGvHD, causing impaired thymopoiesis ( **A**), with reduced thymic export and a distorted T cell receptor (TCR) repertoire with potentially autoreactive thymocytes escaping negative selection ( **B**). ECP, by promoting immune tolerance and enabling reduction and cessation of conventional immunosuppression, may allow thymic recovery, resumption of normal thymopoiesis, and complete and long-lasting immunoreconstitution post-haematopoietic stem cell transplantation ( **C**). Abbreviations: Treg, regulatory T lymphocyte; DP, double positive.

Although aGvHD principally involves the skin, gastrointestinal tract, and liver, the thymus is also a primary target, resulting in disruption of thymic architecture with loss of cortico-medullary demarcation, alteration of TEC subpopulations, and depletion of thymocytes ^22– 24^. The precise mechanisms behind aGvHD-induced thymic injury in humans remain incompletely understood, but experimental models have helped delineate the underlying cellular and molecular mechanisms ^22^. TECs are initiators and targets of thymic aGvHD, capable of activating alloreactive donor T lymphocytes independently of APCs, leading to secretion of interferon gamma (IFNγ) and triggering signal transducer and activator of transcription 1 (STAT1)-induced apoptosis of cortical and medullary TECs ^9^. The resulting disruption of architecture and organisation of the thymic microenvironment with thymic atrophy disturbs the normal signalling required for immature thymocyte development, particularly at the triple-negative proliferative stage and with increased apoptosis of double-positive cells ^22, 25, 26^, resulting in impaired lymphopoiesis and reduced thymic export ( Figure 1) ^11, 27^. Acute GvHD also impairs the thymic-independent pathway with reduced expansion of transferred mature donor T lymphocytes, possibly due to loss of peripheral T lymphocyte niches ^28^.

A distorted TCR repertoire is observed in patients with aGvHD ^10^. Disparate donor and recipient major histocompatibility complex (MHC) complexes disturb thymic positive and negative selection, impacting on TCR selection, resulting in thymocytes escaping negative selection, and increasing the survival of autoreactive T lymphocytes ^29– 32^. Thus, aGvHD is detrimental to the quantity and quality of T lymphocyte recovery. The thymus is particularly sensitive to aGvHD, with thymic output being significantly affected, even in grade 1 disease ^11^. Subclinical thymic aGvHD may have an underappreciated adverse effect on the reconstitution of adaptive immunity, causing ongoing infections and incomplete IR post-HSCT.

## Corticosteroid treatment of acute graft-versus-host disease

Corticosteroids, with potent immunosuppressive and anti-inflammatory effects, are the first-line treatment for aGvHD, but a complete response is witnessed in only 25–50% of patients ^33^. Short, intensive courses of corticosteroids induce thymic involution, causing a profound reduction in naïve T lymphocyte production, although with complete recovery following cessation ^34^. However, the precise effects in human thymus and of long-term corticosteroid use are unknown. There is no consensus for second-line therapy for steroid-dependent/-refractory aGvHD, which usually involves the intensification of systemic immunosuppression with a plethora of therapeutic agents that non-selectively target T lymphocytes ^35, 36^. Second-line options include mycophenolate mofetil, anti-tumour necrosis factor alpha antibodies, or mammalian target of rapamycin (mTOR) inhibitors. The use of mesenchymal stromal cells has also been advocated, with mixed success, in part because the product is a cellular therapy and it is difficult to ensure consistency of the cellular content ^37– 39^. Acute GvHD and immunosuppressive treatment concurrently impair thymopoiesis, subjecting patients to further risk of infection, relapse, and development of secondary malignancies, as well as associated toxicity ^40, 41^. A targeted therapy for aGvHD without systemic immunosuppression and that allows thymic recovery is needed ^42^.

## Extracorporeal photopheresis

Extracorporeal photopheresis (ECP) exposes apheresed mononuclear cells to 8-methoxypsoralen and UVA radiation, with re-infusion of photoactivated cells into the patient ^43^. This induces DNA damage and apoptosis of exposed cells, with activated T lymphocytes preferentially affected ^44, 45^. As only 5-10% of lymphocytes are exposed during the procedure, which is insufficient to explain the effects of ECP, it is speculated that the apoptotic cells have indirect immunomodulatory actions on other immunocompetent cells ^43^. These immunomodulatory mechanisms are poorly understood, but generation of regulatory T lymphocytes (Tregs), alteration of cytokine patterns, and modulation of dendritic cells (DCs) appear to be fundamental ^46– 52^.

The modulation of DCs includes increased number due to differentiation of ECP-exposed monocytes ^53, 54^ and stimulation of a DC-tolerogenic state upon phagocytosis of apoptosed cells, characterised by down-regulation of maturation markers and co-stimulatory molecules and increased secretion of anti-inflammatory cytokines, particularly interleukin-10 ^55– 60^. Upon interaction with T lymphocytes, tolerogenic DCs can induce T lymphocyte anergy or apoptosis or stimulate Treg production ^58, 61^. In aGvHD, DCs, as the major APC, present disparate host antigens to donor T lymphocytes, propagating the pathway of cellular injury. Inducing a DC-tolerogenic state and dampening T lymphocyte activation could attenuate the trigger for aGvHD. The modulation of DC number and function may be a central mechanism of ECP. Tregs are essential in maintaining self-tolerance, down-regulating immune responses, and limiting inflammation that may be harmful to the host and contribute to the mechanism of ECP ^62– 67^.

The unique advantage of ECP as a therapy is lack of global immunosuppression but preservation of the graft-versus-leukaemia effect ^68^. Promoting immune tolerance, with selective down-regulation of immune stimulation, could reduce aGvHD and enable a reduction in other immunosuppression, facilitating thymic recovery, restoration of normal T lymphopoiesis, and complete IR ( Figure 1) with improved clinical outcome as ability to fight infections improves and risk of secondary malignancy or relapse diminishes. It is well tolerated with few adverse effects, and reports of clinical efficacy are impressive ^69– 77^. Whilst the immune-sparing effects of ECP have been demonstrated ^78, 79^, further randomised controlled studies are needed as well as investigation of the effect of ECP on thymic recovery. Further elucidation of the underlying mechanisms at play, as well as the optimal treatment schedule, is required to ascertain fully the role of ECP in aGvHD treatment.

## Abbreviations

aGvHD, acute graft-versus-host disease; APC, antigen-presenting cell; CDR3, complementarity determining region 3; DC, dendritic cell; ECP, extracorporeal photopheresis; HSCT, allogeneic hematopoietic stem cell transplant; IFNγ, interferon gamma; IR, immunoreconstitution; MHC, major histocompatibility complex; mTOR, mammalian target of rapamycin; NK, natural killer; STAT1, signal transducer and activator of transcription 1; TCR, T cell receptor; TEC, thymic epithelial cell; TREC, T cell receptor excision circle; Treg, regulatory T lymphocyte.
